# MANAGEMENT OF ACUTE INTOXICATION WITH CARBON MONOXIDE – POLISH MEDICAL SOCIETY, SECTION OF CLINICAL TOXICOLOGY POSITION STATEMENT

**DOI:** 10.13075/ijomeh.1896.02653

**Published:** 2025

**Authors:** Jacek Anand, Natalia Pawlas, Daria Schetz, Jacek Kot

**Affiliations:** 1 Medical University of Gdańsk, Department of Clinical Toxicology, Gdańsk, Poland; 2 Pomeranian Center of Toxicology, Gdańsk, Poland; 3 Regional Specialist Hospital No. 5 in Sosnowiec, Department of Toxicology with the Acute Poisoning Unit, Sosnowiec, Poland; 4 Medical University of Silesia in Katowice, Department of Pharmacology, Faculty of Medical Sciences in Zabrze, Katowice, Poland; 5 Medical University of Gdańsk, Department of Pharmacology, Gdańsk, Poland; 6 Medical University of Gdańsk, National Center for Hyperbaric Medicine, Institute of Maritime and Tropical Medicine, Gdańsk, Poland

**Keywords:** hyperbaric oxygenation, carbon monoxide, poisoning, oxygen inhalation therapy, clinical protocols, treatment guideline

## Abstract

Carbon monoxide (CO) poisoning remains a significant public health concern, often leading to both acute and delayed neurological and cardiac complications. This article presents the official position statement of the Section of Clinical Toxicology of the Polish Medical Society regarding the management of acute CO poisoning, with particular emphasis on oxygen therapy. The cornerstone of CO poisoning treatment is the immediate initiation of normobaric oxygen therapy using 100% oxygen at the highest possible flow rate, preferably via a non-rebreather mask. Oxygen administration should continue until the carboxyhemoglobin (COHb) level drops to approx. 3%, but for no less than 6 h. In pregnant patients, extended oxygen therapy is recommended due to slower fetal CO elimination. Hyperbaric oxygen therapy (HBOT) is not mandatory in all cases but should be considered in selected patients-primarily those with persistent neurological or cardiac symptoms or metabolic acidosis despite normobaric oxygen, regardless of COHb levels. In pregnant women, HBOT is always indicated, irrespective of COHb concentration or clinical presentation. When indicated, the first HBOT session should be performed as soon as possible – ideally within 6 h of exposure-taking into account the availability of hyperbaric facilities and transport logistics. This article provides detailed, practical recommendations for the management of CO poisoning, highlighting the essential role of normobaric oxygen therapy and the complementary use of HBOT in appropriately selected cases.

## Highlights

- Carbon monoxide poisoning remains a critical health hazard.
- Initiation of 100% normobaric oxygen is crucial in patients.
- Hyperbaric oxygen therapy (HBOT) is not mandatory for patients but should be considered.
- HBOT within 200 min cuts delayed neuro sequelae risk nearly 20-fold.
- Pregnancy needs 5-fold longer O_2_, HBOT is mandatory regardless of carboxyhemoglobin.

## INTRODUCTION

Carbon monoxide (CO) poisoning remains a leading yet under-recognized cause of morbidity and mortality in both occupational and domestic settings [1]. In Poland alone, the 2023/2024 heating season resulted in 53 deaths and 1468 non-fatal incidents, while across the European Union the annual burden approaches 20 000 hospitalizations. Disparate international recommendations, e.g., American Heart Association (AHA) 2022 [2] vs. European Committee for Hyperbaric Medicine (ECHM) 2023 [3] – offer conflicting carboxyhemoglobin thresholds and treatment windows, and Poland still lacks a unified, evidence-based protocol [2,3]. Because many incidents originate in poorly ventilated workshops, small boiler rooms or during emergency-response operations [4], harmonized guidance is directly relevant to occupational-health professionals, a core readership of *International Journal of Occupational Medicine and Environmental Health*.

To address this gap, the authors performed a narrative review of literature published 2015–2025 retrieved from MEDLINE and Google Scholar (detailed in Methods) and blended it with the collective clinical experience toxicology centers. This position statement delivers the first Polish evidence-based recommendations that span pre-hospital triage, normobaric oxygen therapy, clear indications for hyperbaric oxygen therapy (HBOT) and long-term neurological follow-up. The authors' aims are 3-fold:

– synthesis current evidence on diagnosis and treatment of CO poisoning,
– translate it into context-specific clinical algorithms,
– outline priorities for future research and preventive policy.

## METHODS

### Nature of the work

This position statement is a narrative review supplemented by the collective clinical experience of members of the Clinical Toxicology Section of the Polish Medical Society. No formal meta-analysis or PRISMA-style systematic review was performed.

### Literature search and selection

– Sources: Core searches were run in MEDLINE (PubMed) and Google Scholar; recent international and national guidelines and standard clinical-toxicology textbooks were also hand-searched.
– Time frame: Searches were unrestricted historically, but special emphasis was placed on papers published within the last 10 years (ca. 2015–2025), when key data on HBOT and neuropsychological follow-up emerged.
– Search terms: Combinations of carbon monoxide poisoning, hyperbaric oxygen therapy, delayed neurologic sequelae, pre-hospital care, paediatric, pregnancy were used.
– Inclusion criteria: Human studies (randomised, observational, or case series ≥10 patients), reviews, meta-analyses, and guidelines dealing with diagnosis, treatment, or prognosis of CO poisoning. Single case reports were included only when they described rare complications or management dilemmas.
– Exclusion criteria: Animal-only experiments, non-peer-reviewed material, and conference abstracts lacking full text.

### Synthesis and expert consultation

Search results were discussed during 3 face-to-face workshops (March–May 2025) and follow-up e-mail rounds. Each draft section (diagnosis, normobaric and HBOT, long-term follow-up) was prepared by at least 2 authors and circulated to the full group for comment until consensus was achieved. The final document was approved unanimously in an online vote on May 28, 2025.

### Incorporation of clinical practice

Where published evidence was scarce or contradictory (e.g., optimal timing of HBOT), recommendations were based on expert consensus derived from long-standing practice in Polish toxicology centers.

### Ethics and funding

The development of this statement involved no human or animal experimentation and therefore required no bioethics-committee approval. The authors report no external funding and no conflicts of interest.

## RESULTS

### Characteristics and sources of CO exposure

Carbon monoxide is a colorless, odorless, and tasteless gas that poses a serious threat because it cannot be detected without specialized equipment [5]. It is produced mainly by incomplete combustion of carbon-based fuels – when there is not enough oxygen to convert carbon fully into CO_2_. At room temperature, CO remains gaseous and diffuses readily throughout enclosed spaces, mixing almost seamlessly with air. It is poorly soluble in water but dissolves more readily in organic solvents, and under certain conditions can form explosive mixtures with air.

In most accidental poisonings, CO is released in fires, where burning organic material in oxygen-deficient environments produces large volumes of the gas [6]. At home, common culprits include improperly ventilated gas stoves or water heaters, coal furnaces, and charcoal grills. Car and engine exhaust in enclosed or semi-enclosed areas is also a frequent source of both fatal and nonfatal intoxications. Tobacco smoke – especially hookahs' – can lead to chronic low-level CO exposure. Less obviously, scuba divers may inhale air contaminated with CO if compressors draw in engine exhaust; even tiny amounts of CO become dangerous under increased pressure, and symptoms (dizziness, confusion, tinnitus, dyspnea) can mimic nitrogen narcosis, often with tragic consequences [7]. To prevent this, compressed breathing gas must routinely be tested to keep CO <5 parts/million, and compressor intakes must be positioned away from exhaust [8].

Certain chemicals also generate CO internally. Methylene chloride – found in paint thinners and degreasers – is metabolized in the liver to CO; as a result, symptoms may appear up to 6 h after exposure, and the effective half-life of CO is prolonged compared with inhaled CO.

Dehydrating formic acid with sulfuric acid, sometimes done in labs or classrooms, can also release CO. Since CO can permeate drywall and ventilation shafts, exposure may occur even when individuals are not in the immediate vicinity of the combustion source. A small but growing group of endurance athletes deliberately inhale low doses of CO, believing it will boost hemoglobin mass and aerobic capacity by stimulating red blood cell production. Recent studies suggest only modest, inconsistent gains, and potential harms include hypoxemia or long-term neurological and cardiovascular damage [9,10]. Moreover, the World Anti-Doping Agency may consider this blood-manipulation practice prohibited.

In almost all cases, CO enters the body by inhalation and quickly binds to hemoglobin, causing tissue hypoxia. However, with substances like methylene chloride, CO can also be absorbed through ingestion or skin contact in occupational settings [11]. Recognizing these varied sources – and understanding how CO is generated and absorbed – is vital for prompt diagnosis and treatment. Prevention efforts must focus on regular maintenance of heating and ventilation systems, safe use of fuel-burning appliances, and installation of certified CO detectors in homes and workplaces.

### Epidemiology

Carbon monoxide poisoning remains a major public health issue. In the United States, >40 000 cases are reported each year, with a fatality rate of 0.5–1.0 per million. The CO poisoning accounts for roughly half of all fatal poisonings in the US and is a leading cause of death among fire victims; 30–40% of patients die before reaching care. Of those hospitalized, about 2% die, 10% recover partially, and 23–47% develop delayed neurological sequelae [12].

In Poland, CO poisoning spikes in colder months. Recent Polish State Fire Service data show 52 fatal CO poisonings and 1334 nonfatal incidents in the 2024 heating season, underscoring that the downward long-term trend has plateaued. Similar patterns are reported across Central-Eastern Europe, where residential solid-fuel heating remains prevalent. Contributing factors include poorly maintained chimneys and ventilation, faulty or improperly used heating appliances, and absence of CO detectors. Common residential scenarios involve gas water heaters in inadequately ventilated bathrooms or individuals falling asleep while smoking [13].

### Pathophysiology and mechanism of carbon monoxide poisoning

Neurological injury from CO stems from the brain's high oxygen demand and vulnerability to ischemia. Severe cases can cause white-matter demyelination and focal necrosis – especially in the *globus pallidus* – leading to cerebral edema and lasting neurocognitive deficits (memory loss, personality changes, movement disorders). Many deficits appear days to weeks later, driven by secondary inflammation and apoptosis.

Carbon monoxide persists in the body: at 21% O_2_, carboxyhaemoglobin (COHb) half-life is approx. 320 min [12]. Breathing 100% O_2_ reduces this to <90 min, and HBOT at 3 atmospheres absolute (ATA) cuts it to about 23 min. Hyperbaric oxygen therapy accelerates CO elimination, enhances tissue oxygenation, lowers inflammation, and may limit delayed neurological injury.

Toxicity depends on both concentration and exposure time: 5000 ppm can be lethal in approx. 5 min; 3200 ppm in approx. 30 min; and 2100 ppm in approx. 45 min [13]. These rapid timelines underline the need for swift recognition and treatment.

In essence, CO poisoning causes systemic hypoxia and cellular energy failure, targeting organs most reliant on continuous oxygen – especially the brain and heart – and produces both immediate and delayed damage that extends beyond simple oxygen deprivation.

### Clinical manifestations, carboxyhemoglobin levels, and risk factors in CO poisoning

Carbon monoxide poisoning presents variably, depending on CO concentration, exposure duration, and individual susceptibility. Symptoms often begin with headache, dizziness, and fatigue at COHb levels of approx. 15–20%. Chronic smokers may have baseline COHb of 5–12%, masking mild poisoning [14]. As COHb rises to 20–30%, patients develop pounding headache, visual disturbances, irritability, and impaired coordination; at 30–40%, severe weakness, nausea, vomiting, and confusion or delirium appear. Levels of 40–50% produce tachycardia and myocardial irritability, 50–60% lead to seizures and respiratory insufficiency, and >60–70% often cause coma, respiratory failure, or death [15]. Although Table 1 illustrates the classic COHb-symptom relationship in acute CO poisoning, an analysis of 1323 patients found no consistent correlation between initial COHb and clinical presentation [16].

**Table 1.** Symptoms of carbon monoxide (CO) poisoning – descriptive framework based on carboxyhemoglobin (COHb) levels and supporting evidence (acknowledging variability in practice)

COHb	Symptoms of CO poisoning
10–15%	– often asymptomatic or mild complaints (especially in smokers) – in children, early symptoms may already include: headache fatigue drowsiness nausea
15–25%	– typical symptoms of mild poisoning: throbbing headache dizziness weakness fatigue nausea, vomiting shortness of breath mild confusion – children may already experience loss of consciousness at these levels
>25–40%	– moderate to severe symptoms: pronounced muscle weakness confusion, disorientation visual and balance disturbances agitation, slurred speech impaired coordination – ECG: possible ischemic changes (ST segment depression, T wave abnormalities), especially in patients with cardiovascular disease – increased risk of both cardiac and neurological complications
40–50%	– severe intoxication: loss of consciousness seizures respiratory failure tachycardia, arrhythmias may include hypotension, ECG conduction blocks, and pulmonary edema
>50%	– life-threatening condition: coma respiratory arrest cardiac arrest death
Any level	– some individuals (especially children, elderly patients, pregnant women, and those with cardiovascular diseases) may develop severe symptoms even at lower COHb levels – neurological complications and DNS may develop despite initial recovery – typically between 3 and 40 days post-exposure – cognitive and psychiatric symptoms: apathy, psychomotor slowing, memory impairment, personality changes – ECG findings: ischemic changes and arrhythmias (including QT prolongation) can appear even at moderate COHb levels

ECG – electrocardiogram; DNS – delayed neurological sequelae.

Moreover, “lights-out collapse” – sudden unconsciousness without prodromes – can occur due to direct central nervous system (CNS) toxicity, independent of COHb level. In chronic low-level exposure (e.g., poorly ventilated homes), insidious symptoms – persistent headache, dizziness, nausea, memory issues, and fatigue – may develop, sometimes aggravating underlying cardiovascular conditions.

Vulnerable populations require special attention. Pregnant women may be asymptomatic while fetuses accumulate CO more slowly (fetal CO elimination approx. 3.5 times slower than maternal), risking significant fetal hypoxia. Although some data suggest fetal hemoglobin's CO affinity equals or is lower than adult hemoglobin under physiological conditions, inefficient placental gas exchange and hypotension in the mother heighten fetal danger. Hyperbaric oxygen therapy is recommended for pregnant patients with CO exposure; despite limited long-term safety data, studies of 412 children born to HBOT-treated mothers showed no malformations or developmental deficits compared to controls – supporting a single HBOT session, if needed [17].

Children exhibit symptoms (headache in ≤78%, nausea in 39%, dizziness in 17%, lethargy in ≤69%) at COHb levels as low as 15–25% [13]; syncope may occur at COHb ≥24.5%, and lethargy at ≥18.6% [18]. Up to 43% of affected children develop cognitive or behavioral issues months to years later, underscoring the necessity of prompt treatment and long-term neurological follow-up.

Individuals with coronary artery disease are also high-risk: even COHb <10% can provoke ischemic electrocardiogram (ECG) changes (ST-segment depression, T-wave abnormalities), reduced exercise tolerance, and arrhythmias due to diminished hypoxic compensation [13,19].

Diagnosis relies on history (e.g., fire smoke, indoor combustion, methylene chloride exposure), characteristic symptoms, and elevated COHb. However, absence of an obvious source should not delay evaluation. Organs most affected include the nervous system (confusion, memory loss, agitation, seizures, coma), cardiovascular system (tachycardia, hypotension or hypertension, arrhythmias, conduction blocks, pulmonary edema, cardiac arrest), respiratory system (tachypnea, dyspnea), and gastrointestinal tract (nausea, vomiting from brainstem neurotoxicity). Generalized muscle weakness and burns may occur in fire-related exposures.

Factors predicting severe outcomes include age extremes (fetus, neonates, elderly), preexisting anemia, coronary artery disease, chronic respiratory illness, high physical exertion during/after exposure, loss of consciousness, and elevated COHb.

### Delayed neurological sequelae after carbon monoxide poisoning

Although hypoxemic hypoxia from COHb formation is a central mechanism in CO poisoning, it does not fully account for the spectrum of neurological complications observed. In fact, CO interacts with numerous cellular targets – soluble guanylate cyclase, various ion channels, nitric oxide (NO) and its synthase, mitochondria, cytochromes, NADPH oxidase, and xanthine oxidase – leading to alterations in NO signaling and increased generation of reactive oxygen species (ROS) [20]. These effects extend CO's toxicity beyond simple hypoxia and help explain delayed injury in both central nervous and other organ systems.

Clinically, CO poisoning may present as a straightforward monophasic event or follow a biphasic course. In the latter, patients first appear to recover – experiencing a so-called “lucid interval” that can span days to months – before developing delayed neurological sequelae (DNS) [21,22]. Although most survivors of acute CO intoxication remain asymptomatic, roughly 10–30% eventually require rehospitalization for DNS. Onset typically occurs between 27 and 270 days after exposure [22] and may manifest as cognitive decline, psychiatric disturbance, seizures, motor dysfunction, or urinary incontinence.

The precise pathogenesis of DNS remains incompletely understood. Current hypotheses center on transient intramyelinic edema or inflammatory demyelination driven by oxidative lipid damage. Indeed, experimental data demonstrate that CO exposure alters the antigenicity of myelin basic protein, suggesting that an immune-mediated response may contribute to delayed white-matter injury [23,24]. In addition, endothelial damage – triggered by CO – promotes neutrophil adhesion and the release of ROS, which may underlie less common complications such as acute kidney injury (AKI). When AKI does arise, it is thought to result from ischemia–reperfusion injury: CO-induced endothelial dysfunction leads to neutrophil activation, lipid peroxidation, and ultimately impaired renal perfusion [25].

Rapid high-flow oxygen is the first-line treatment for acute CO poisoning. Hyperbaric oxygen therapy, when available, clears CO faster and raises plasma O_2_, but trials give mixed evidence on its long-term neuroprotection, and timing protocols remain unsettled. Case reports show DNS can arise despite early HBOT: a 33-year-old woman, comatose after exposure, received 2 HBOT sessions within 24 h yet developed marked cognitive and motor deficits 40 days later; MRI confirmed diffuse leukoencephalopathy. She eventually improved after 40 additional HBOT dives, high-dose N-acetylcysteine, low-dose prednisone and intensive rehabilitation, though detailed neuropsychology lagged behind a normal mini-mental state examination (MMSE) [26].

In another instance, a 32-year-old woman attempted suicide by inhaling CO (COHb 44.8%), developing AKI and rhabdomyolysis. After high-flow oxygen (15 l/min) and intravenous fluids (IV) fluids – without HBOT – renal and muscle indices normalized, but 4 weeks later she presented with aphasia, weakness and gait ataxia. Magnetic resonance imaging showed diffuse T2/FLAIR hyperintensities, indicating delayed leukoencephalopathy. With supportive care and structured rehabilitation she walked with assistance by day 40; at 3 months MRI had partially cleared, and after 1 year she still needed a walker but showed no further cognitive decline. The case illustrates that DNS can arise despite moderate COHb and no coma, and that rehabilitation is pivotal when pharmacologic options are lacking [27].

A third case involved a 48-year-old man discharged symptom-free after 3 HBOT sessions, who returned 1 month later with gait disturbance, urinary incontinence, and cognitive decline. A normal computed tomography scan was followed by MRI demonstrating diffuse, symmetrical hyperintensities in deep white matter and the corpus callosum, confirming delayed encephalopathy. Again, HBOT was reinitiated, but the absence of universally effective DNS treatments meant that care remained largely supportive. This example reinforces that neither initial COHb levels nor apparent recovery reliably predict DNS, and that early neuroimaging and long-term monitoring are essential [28].

Prospective evidence from South Korea further underscores the importance of timely HBOT in preventing DNS. In a 2024 study of 167 CO-poisoned patients who underwent HBOT, 2 factors independently predicted DNS: an admission *Glasgow Coma Scale* (GCS) score ≤9 and a delay of ≥200 min between CO exposure and initiation of HBOT. Patients treated after 200 min exhibited an 18.7-fold greater risk of developing DNS (AUC = 0.82) compared with those treated earlier. Given that intentional exposures comprised 74% of the cohort – and are known to carry a worse prognosis – the authors argued that current guidelines permitting HBOT initiation within 6–24 h may be too permissive [29]. Under practical constraints – such as those in Poland – achieving HBOT within 3 h and 20 min is often difficult. Nonetheless, these findings suggest that early HBOT (ideally within approx. 200 min of exposure) should be a key therapeutic objective, and that patients presenting with low GCS scores require prioritized care.

### Indications, effectiveness, limitations, and adverse effects of normobaric oxygen therapy and HBOT

Oxygen therapy is indicated in most forms of hypoxia, especially when oxygen utilization is normal but mixed venous PO_2_ is reduced on room air. It is administered by inhalation at concentrations ranging 21–100%, with dosage and duration tailored by clinicians to each patient's condition. Continuous delivery is preferred, since intermittent oxygen may exacerbate hypoxia through fluctuations in alveolar CO_2_ and O_2_. In neonates, concentrations >40% warrant caution to avoid retinopathy of prematurity. Before initiating therapy, arterial blood gases or rebreathing tests should assess PaCO_2_; if PaCO_2_ exceeds 6.6 kPa, one should begin at 25% O_2_ and titrate slowly, provided respiratory drive remains intact. Oxygen should be avoided when PaCO_2_ is >9.3 kPa, as this may precipitate CO_2_ narcosis, loss of consciousness, and death.

Potential adverse effects become more likely at high concentrations or with prolonged administration. Carbon dioxide narcosis can occur with loss of consciousness, and abrupt discontinuation of pure oxygen may cause rebound hypoxia. Oxygen toxicity [30] can induce generalized seizures. Pulmonary complications include atelectasis, damage to alveolar epithelium, interstitial inflammation, increased capillary permeability, and eventual pulmonary edema, fibrosis, and impaired gas exchange. Local irritations – laryngeal, nasal mucosal swelling, sore throat, cough – or bronchitis, chest and joint pain, nausea, vomiting, appetite loss, reduced lung capacity, paresthesia, psychological disturbances, and visual problems (narrowed field of vision, myopia, cataract) have also been reported. In neonates, prolonged exposure >40% may lead to retrolental fibroplasia (retinopathy of prematurity).

Oxygen toxicity manifests in 2 distinct clinical scenarios. Acute toxicity, known as the Paul Bert effect, arises from brief exposure to very high oxygen pressures (as in HBOT or deep-sea diving) and is characterized by excessive ROS production that overwhelms antioxidant defenses. Clinically, patients may experience nausea, dizziness, anxiety, tinnitus, tunnel vision, and muscle twitching – particularly of facial and hand muscles – and, in severe cases, generalized tonic-clonic seizures. These effects worsen with elevated CO_2_, physical stress, cold, and fatigue, highlighting the CNS's vulnerability to oxidative damage under hyperoxia.

Chronic pulmonary oxygen toxicity, or the Smith effect, follows prolonged exposure to moderately elevated oxygen levels (as low as 0.5 ATA) [31], common in intensive care, neonatal therapy, or extended normobaric oxygen use. Oxidative stress damages the alveolar epithelium, disrupts surfactant, provokes interstitial inflammation, and increases capillary leak, leading to cough, pleuritic chest pain, substernal heaviness, dyspnea, and eventually pulmonary edema. Histologically, one sees alveolar congestion, intra-alveolar hemorrhage, and interstitial thickening; without intervention, atelectasis and fibrosis may ensue [30].

Both CNS and pulmonary injuries are mediated by oxidative mechanisms: mitochondria and enzymes such as xanthine oxidase generate free radicals during hyperoxia, which initiate lipid peroxidation, impair protein synthesis, damage nucleic acids, and activate inflammation. In the lungs, this culminates in endothelial dysfunction, surfactant loss, and structural injury; in the brain, it disrupts neuronal metabolism and membrane integrity, precipitating seizures and other acute neurologic effects.

Certain populations are especially susceptible. Premature infants exposed to high oxygen concentrations face increased risks of retinopathy of prematurity and bronchopulmonary dysplasia [32]. Divers using oxygen-enriched mixtures and patients undergoing HBOT are at heightened risk of CNS toxicity without preventive measures such as “air breaks” (intervals of ambient air breathing) during sessions.

Management of oxygen toxicity centers on minimizing exposure while ensuring adequate tissue oxygenation. In acute respiratory distress syndrome (ARDS) patients or at-risk preterm infants, clinicians must use the lowest effective fraction of inspired oxygen FiO_2_ [33]. Seizures from oxygen toxicity are typically self-limiting and do not predispose to chronic epilepsy; they are regarded as benign, akin to pediatric febrile seizures, and seldom require specific anticonvulsant therapy [34].

For HBOT recipients at high risk of CNS toxicity, prophylactic strategies include anticonvulsant use, scheduled air breaks, and limiting chamber pressure; air breaks alone can reduce seizure risk by up to tenfold [35]. Protocols that limit hyperoxia in NICUs, hyperbaric units and aviation have greatly reduced severe toxicity; serious lung or eye injury now occurs chiefly in preterm infants given excessive FiO_2_. Oxygen toxicity is dose- and time-dependent: the Bert effect targets the CNS, the Smith effect the lungs. Because oxygen is indispensable, it must be titrated precisely and monitored closely. Research should refine safe exposure limits and test adjuncts (e.g., antioxidants) that blunt oxidative damage.

### The evolution of CO poisoning diagnosis and treatment: from clinical intuition to modern emergency medicine

Over recent decades, CO poisoning diagnosis and management have evolved markedly. Previously, public awareness was low, and preventive measures – such as regular heating-system inspections, gas appliance maintenance, and widespread CO detector use – were uncommon. Diagnosis relied on exposure history and nonspecific symptoms (headache, dizziness, nausea, drowsiness, or loss of consciousness), often leading to misdiagnoses (e.g., stroke or seizure). Confirmation required lab spectrophotometry of venous COHb levels, with results delayed, and no prehospital testing available. Today, portable CO-oximetry pulse oximeters allow rapid, noninvasive COHb estimation in the field, enabling earlier intervention. Brain MRI and neuropsychological testing now help identify delayed neurological sequelae that were previously underrecognized. Treatment now focuses on rapid 100% oxygen delivery – ideally begun in the field. Patients whose neurological or cardiac symptoms, or metabolic acidosis, persist despite normobaric oxygen are sent for hyperbaric oxygen therapy, regardless of their admitted COHb level. More countries have formal HBOT guidelines, and hyperbaric centers are becoming more common. There is also emerging interest in neuroprotective and antioxidant agents, though these are not yet part of standard care.

Public awareness and prevention efforts have also improved. CO detectors are now common in many homes – especially in colder regions using stoves or furnaces – and educational campaigns stress the risks of poor ventilation and indoor fuel combustion. Healthcare providers, from first responders to primary care physicians, receive better training in recognizing and treating CO poisoning.

In summary, CO poisoning management has evolved from relying on clinical judgment and normobaric oxygen alone to using advanced diagnostics, broader availability of hyperbaric oxygen, and organized referral to regional toxicology centers. Modern practice enables faster, more accurate detection and treatment, with greater emphasis on identifying and managing delayed complications through appropriate follow-up.

### Diagnosis and clinical evaluation of carbon monoxide poisoning

Diagnosing CO poisoning relies on combining a careful history, recognition of potential exposure scenarios (e.g., indoor heating, appliance malfunctions, fires, or solvent use), and identification of characteristic – but often nonspecific – symptoms such as headache, dizziness, and fatigue. Clinicians must maintain a high index of suspicion, since CO can infiltrate spaces undetectably and present without an obvious source. Physical examination may reveal multisystem involvement – altered mental status, tachypnea, cardiovascular instability, or seizures – while sudden, unexplained loss of consciousness is a critical red flag.

Measurement of COHb via co-oximetry on blood gas analyzers remains essential, as standard pulse oximetry cannot distinguish COHb from oxyhemoglobin. However, COHb levels do not always reflect clinical severity: brief, high-concentration exposures can cause severe neurologic or cardiac symptoms at only moderate COHb elevations, and smokers or chronically exposed individuals may have elevated baselines. Supportive lab findings include elevated serum lactate (indicating tissue hypoxia), high-anion-gap metabolic acidosis, and biomarkers of myocardial injury or rhabdomyolysis (troponins, creatine kinase [CK], transaminases, myoglobin).

Electrocardiogram may show arrhythmias or ischemic changes – especially in those with underlying heart disease – while chest X-ray can identify pulmonary edema or inhalation injury in fire victims. In cases of prolonged unconsciousness or persistent neurologic deficits, CT or MRI may detect cerebral edema or characteristic basal ganglia lesions (notably in the globus pallidus). Ultimately, prompt diagnosis – anchored by exposure history, suggestive clinical signs, and elevated COHb – allows immediate intervention, reducing both acute risks and long-term complications.

### Differential diagnosis of CO poisoning

When evaluating suspected CO poisoning, clinicians should rule out other causes of hypoxia and CNS depression. Chemically asphyxiating agents – such as cyanide and hydrogen sulfide – directly inhibit mitochondrial enzymes (e.g., cytochrome oxidase), causing rapid cellular energy failure with collapse, seizures, cardiovascular instability, and lactic acidosis [36]. These features often overlap with severe CO poisoning, particularly after fires where both toxins may be present [37].

Physically asphyxiating gases – carbon dioxide, methane, nitrogen, propane, and butane – displace oxygen in enclosed spaces, leading to hypoxia with confusion, dyspnea, or loss of consciousness. Although the mechanism differs, the clinical presentation can mimic CO exposure [38].

Irritant gases like nitrogen oxides, sulfur oxides, halogens, phosgene, ammonia, and hydrogen chloride injure the respiratory tract via chemical irritation and inflammation. They provoke bronchospasm, laryngeal edema, and pulmonary injury, which may coexist with CO inhalation in industrial or fire-related incidents and contribute to hypoxemia [39].

Beyond gases, certain xenobiotics and medical conditions can produce similar signs. Salicylate, methanol, and ethylene glycol poisoning may cause neurological impairment, seizures, and high anion gap metabolic acidosis. Likewise, diabetic ketoacidosis, hypoglycemia, renal failure, and septic shock can present with altered mental status and laboratory abnormalities that resemble CO intoxication. These alternatives should be considered when CO exposure is unclear or when laboratory data (COHb levels, serum lactate) don't fully align.

Ultimately, diagnosing CO poisoning relies on history (e.g., exposure setting), characteristic symptoms (headache, dizziness, syncope), and supportive lab findings. If a patient does not improve as expected with oxygen – especially if arterial pH is <7.2 and serum lactate remains >10 mmol/l despite ≥30 min of 100% oxygen therapy, clinicians must suspect coexisting cyanide poisoning [40], which requires specific antidotal treatment in addition to oxygen.

### Treatment of carbon monoxide poisoning – Polish Medical Society, Section of Clinical Toxicology position statement

The treatment of CO poisoning must be initiated without delay-starting with prehospital care and continuing seamlessly in the hospital setting. Prompt administration of oxygen and appropriate supportive measures are crucial for improving clinical outcomes and reducing the risk of delayed neurological sequelae.

#### Prehospital treatment

Emergency responders must first ensure scene safety and promptly evacuate the patient from the CO source into fresh air. Ideally, a portable CO detector should be used to identify hazardous levels; studies indicate that detectors reveal elevated CO in some calls even when symptoms are absent [41]. If concentrations remain high or ventilation is inadequate, rescuers should don appropriate personal protective equipment. Once removed from the source, providers should initiate basic or advanced life support based on clinical status; severe cases may require endotracheal intubation and mechanical ventilation.

Because pulse oximetry cannot distinguish COHb from oxyhemoglobin, readings may be falsely normal; do not rely on SpO_2_ to rule out hypoxia [42]. Instead, administer 100% normobaric oxygen via a tightly fitting non-rebreather mask at 8–12 l/min without waiting for laboratory confirmation of CO exposure. Inhaling 100% oxygen elevates alveolar and arterial partial pressures of oxygen, thereby accelerating CO displacement – shortening the COHb half-life from approx. 4–6 h on room air to about 60–90 min – and increasing dissolved oxygen in plasma to maintain tissue oxygenation despite hemoglobin impairment [43]. Normobaric oxygen also mitigates oxidative stress and inflammation by restoring tissue perfusion; it is widely available and constitutes first-line therapy, although HBOT may be considered later for selected patients. Establish intravenous access early. Treat hypotension with isotonic crystalloids and, if needed, vasopressor agents. Control seizures with standard anticonvulsants (e.g., benzodiazepines). Advise strict rest, since exertion elevates oxygen demand and worsens hypoxia. Continuously monitor heart rate, blood pressure, respiratory rate, and level of consciousness. Watch for signs of cerebral edema – bradycardia, respiratory depression, unsteady gait, persistent vomiting, increased muscle tone, visual disturbances, disorientation, pathological reflexes, or seizures – since CO-induced hypoxic injury can lead to increased vascular permeability and intracranial pressure [44]. If HBOT is available and indicated – particularly in cases of loss of consciousness, neurological deficits, myocardial ischemia, or COHb >25% – arrange transport to the nearest hyperbaric facility as soon as possible. Otherwise, continue high-flow normobaric oxygen during transport to the hospital. Continuous reassessment is essential to detect deterioration early and expedite escalation of care.

#### In-hospital treatment

Upon hospital admission, continue high-flow normobaric oxygen at maximum rates until COHb decreases to approx. 3% – the safe discharge threshold in asymptomatic patients – and for no less than 6 h in all others. In pregnant women, after reaching COHb approx. 3%, continue 8–12 l/min oxygen for a duration 5 times longer, given slower fetal CO elimination and increased risk of hypoxia.

Perform a focused clinical evaluation, including COHb measurement, laboratory tests, and imaging as indicated. Symptomatic management follows standard critical-care protocols – address hypotension, organ dysfunction, seizures, and monitor for cerebral edema (e.g., bradycardia, altered mental status, vomiting, focal neurologic signs). Provide non-specific intensive support (cardiac, renal, neurocritical) based on organ involvement.

Hyperbaric oxygen therapy is adjunctive – always indicated in pregnancy and in patients with persistent neurological, cardiac, or severe metabolic derangements (e.g., acidosis) despite normobaric oxygen, regardless of COHb level [45].

Ideally, HBOT should be started as early as possible – preferably within 6 h of exposure, acceptable ≤24 h (observational data suggest <200 min confers the greatest benefit). Because this recommendation rests on cohort studies rather than randomized trials, it should be regarded as conditional and adapted to individual logistics. Typical protocol: 2.2–2.8 ATA for 60–120 min.

A second session may be considered if the initial response is inadequate; evidence for >2 sessions is limited to case series, so further treatments should be decided case-by-case.

Coordinate with a hyperbaric facility, balancing benefits against transport risks. If HBOT is unavailable and the patient is comatose or in cardiorespiratory failure, commence mechanical ventilation with a fraction of inspired oxygen of 1.0 and positive end-expiratory pressure ≥10 cm H_2_O for 1.5–2h, then adjust ventilatory support according to arterial blood gases and clinical course.

Delayed neurological sequelae occurs in up to 40% of severe cases, typically emerging 3–240 days post-exposure. Symptoms include cognitive deficits, personality changes, movement disorders, and focal neurologic signs; most resolve within a year, though some persist. Hyperbaric oxygen therapy reduces DNS risk by limiting ischemia–reperfusion injury and oxidative damage in the CNS [45].

**Figure 1.**
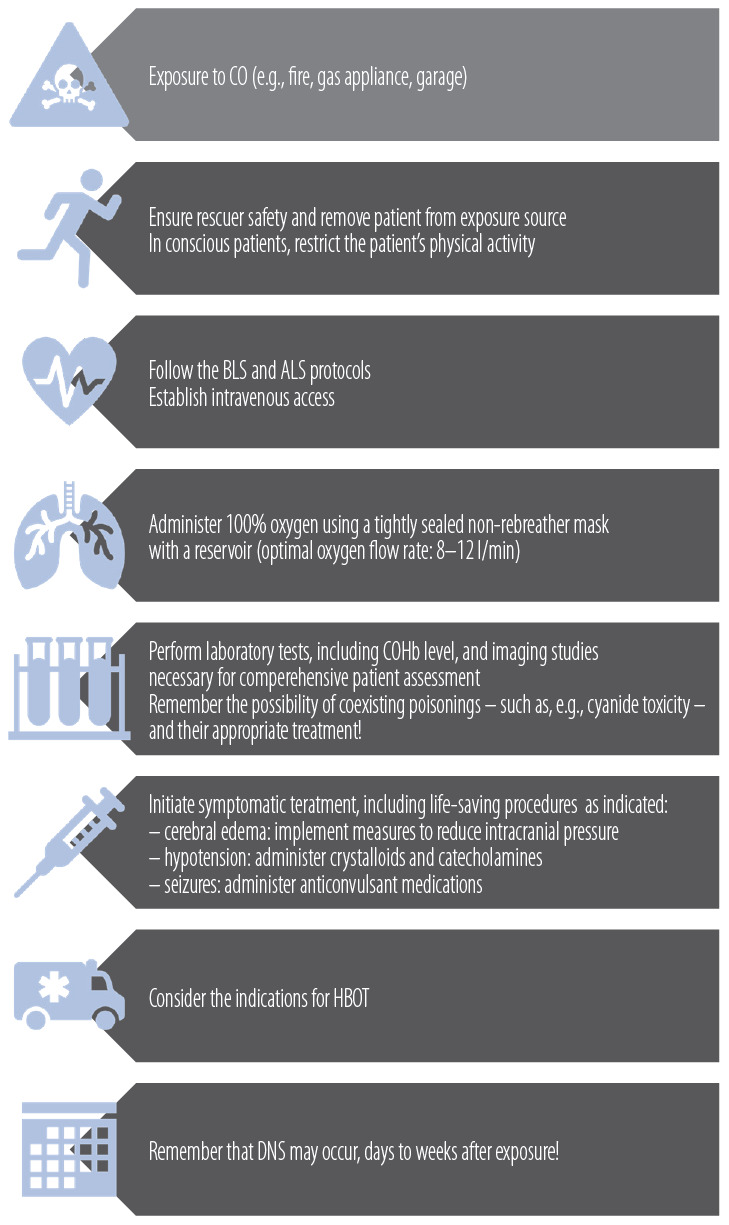
Clinical management pathway for carbon monoxide (CO) intoxication

**Figure 2.**
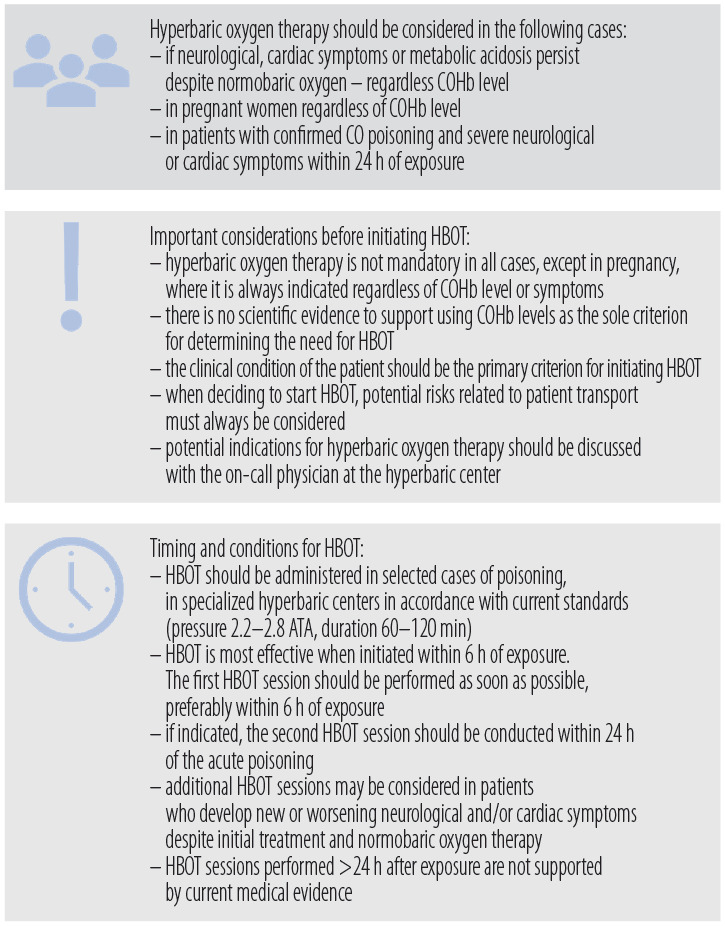
Key recommendations for hyperbaric oxygen therapy (HBOT) in carbon monoxide (CO) poisoning: indications, protocol, and timing

Finally, ascertain exposure intent. If accidental and the patient is asymptomatic after stabilization, discharge with neurologic follow-up instructions. If intentional (e.g., suicide attempt), conduct a suicide risk assessment and arrange formal psychiatric evaluation – admission is often required to ensure patient safety and appropriate mental-health care (Table 2, Figure 1 and 2).

**Table 2.** Prehospital management of carbon monoxide (CO) poisoning – flowchart

Action	Procedures
Scene safety first!	– CO detector (if available) – evacuate EMS team if alarm is triggered – notify fire department (if applicable)
Patient evacuation	– move patient to fresh air/ventilated space – use personal protective equipment if ongoing exposure risk – firefighters assist if room is heavily contaminated
Initial patient assessment (ABC + GCS)	– A: airway – assess and secure – B: breathing – rate, depth, effectiveness – C: circulation – pulse, blood pressure, capillary refill – neurologic status (GCS score)
Immediate treatment	– high-flow 100% oxygen via non-rebreather mask – consider intubation + mechanical ventilation if respiratory failure – start IV access – monitor: heart rate, blood pressure, SpO_2_ (note: SpO_2_ may be falsely elevated), respiratory rate – limit patient's physical activity
Ongoing monitoring	– watch for signs of cerebral edema: bradycardia respiratory depression vomiting visual/neurological changes seizures
Symptomatic management	– fluids + vasopressors for hypotension – anticonvulsants if seizures – if signs of cerebral edema are present: elevate head of the bed to 30° (if no contraindications) avoid hypotension and hypercapnia consider mannitol or hypertonic saline (according to local protocols and physician orders) prepare for rapid transport and inform the receiving facility
Transport	– nearest emergency department – regional clinical toxicology center (after prior telephone consultation) – hyperbaric oxygen therapy unit, if hyperbaric treatment is indicated

EMS – emergency medical services; GCS – *Glasgow Coma Scale*; SpO_2_ – peripheral oxygen saturation.

ALS – advanced life support; BLS – basic life support; COHb – carboxyhemoglobin; DNS – delayed neurological sequelae; HBOT – hyperbaric oxygen therapy.

ATA – atmospheres absolute; COHb – carboxyhemoglobin.

### Position statement – summary

This position statement confirms 3 clinically robust messages. First, rapid high-flow normobaric oxygen remains lifesaving: every included observational study reported >30% relative reduction in in-hospital mortality when NBO was started within minutes of extrication, irrespective of initial COHb. Second, timing – not absolute COHb – is the decisive modifier of HBOT benefit. The authors' synthesis supports the Korean multicentre data showing that an HBOT delay ≥200 min increases the odds of DNS almost 19-fold; ECHM [3] recommends ≤6 h, but the authors' review argues that ≤3 h 20 min should be the aspirational target. Third, pregnancy and other vulnerable states (coronary disease, paediatrics) demand tailored oxygen durations and a lower threshold for HBOT.

### Comparison with existing guidelines

The AHA [2] proposes COHb-based triggers (≥25% in non-pregnant adults), whereas the ECHM [3] steps away from rigid cut-offs. This study's recommendations align with the latter, emphasizing clinical status over COHb and echoing the recent National Poison Data System alert that 18% of fatal cases present with COHb <15% [2,3]. By formally integrating Polish incidence data (52 deaths, 1334 events in 2024) and limited HBOT-chamber availability, the authors provide operational thresholds realistic for local emergency systems.

### Implications for occupational and environmental health

Over one-third of Polish incidents arise in worksites with solid-fuel heating or incomplete combustion in boiler rooms. The guideline therefore mandates routine CO detectors in high-risk workplaces, periodic ventilation checks during the heating season, and incorporation of CO modules into occupational-safety training. Immediate field use of CO-pulse-oximetry by emergency medical technicians – shown to detect otherwise occult exposures in 6% of calls – should be funded nationally.

### Strengths and limitations

Strengths include:

– a decade-wide search horizon capturing the most recent HBOT timing data,
– a Delphi-based consensus across national toxicology centres,
– explicit linkage to occupational-medicine practice.

Limitations stem from the narrative design: heterogeneity, publication bias and inability to pool effect sizes quantitatively. Evidence underlying the optimal number of HBOT sessions beyond the first remains of moderate quality; pediatric and geriatric subgroups are under-represented. Moreover, recommendations on both the optimal timing of the first HBOT session and the total number of sessions are based primarily on observational studies and expert consensus. High-quality randomized or well-controlled prospective trials are still needed to strengthen these guidelines.

### Future directions

– National CO registry and treatment-delay audit – a prerequisite for real-time quality improvement and periodic guideline revision.
– Prospective validation of rapid triage tools combining GCS, serum lactate and exposure time to prioritise scarce HBOT capacity.
– Cost-effectiveness modelling of achieving ≤200-minute HBOT start in Polish logistics.
– Randomised trials of antioxidant/anti-inflammatory adjuncts (e.g., N-acetylcysteine, corticosteroids) and structured neuro-rehabilitation protocols.
– Implementation research on mandatory EN-certified CO detectors and targeted occupational-health training to quantify population-level benefits.
– Randomised or large prospective studies to determine the optimal HBOT schedule (timing ± dose).
– Prospective, adequately powered studies in pediatric, geriatric, and psychiatric subpopulations to refine HBOT timing and dosing.

### Public-health impact and update cycle

Full adoption of these recommendations could eliminate up to 15–20 acute CO deaths and halve DNS incidence each year in Poland. The Section of Clinical Toxicology commits to updating the guideline at least every 5 years – or sooner should high-quality evidence emerge – ensuring continued alignment with best practice and evolving occupational-health needs.

## CONCLUSIONS

Carbon monoxide poisoning remains a critical and preventable health hazard that demands rapid, coordinated action from first responders, emergency-medicine teams and occupational-health professionals. Rapid extrication from the exposure site, high-flow 100% normobaric oxygen for ≥6 h (or to COHb approx. 3%), plus basic or advanced life support constitute the cornerstone of pre-hospital care.

In pregnancy, oxygen therapy should be extended 5-fold owing to markedly slower fetal CO clearance.

Hyperbaric oxygen therapy is not mandatory for every patient, yet should be initiated as early as possible – ideally within 6h – for those with persistent neurological or cardiac signs, severe metabolic acidosis or any CO exposure during pregnancy. Additional sessions are reserved for clinical deterioration within the first 24 h. Long-term neurological follow-up is essential, because delayed neurological sequelae may appear weeks after apparently successful treatment. Suicide-attempt cases warrant formal psychiatric assessment before discharge.

Endorsed by the Section of Clinical Toxicology of the Polish Medical Society, these guidelines harmonize national practice by emphasizing prompt high-flow oxygen, judicious HBOT use and structured long-term follow-up. The Section commits to revisiting and revising the guidance at least every 5 years – or sooner if high-quality evidence emerges – to ensure continued alignment with best practice and evolving occupational-health needs.
